# Using variable importance measures from causal inference to rank risk factors of schistosomiasis infection in a rural setting in China

**DOI:** 10.1186/1742-5573-7-3

**Published:** 2010-07-14

**Authors:** Sylvia EK Sudat, Elizabeth J Carlton, Edmund YW Seto, Robert C Spear, Alan E Hubbard

**Affiliations:** 1 Division of Biostatistics, University of California, Berkeley, USA; 2 Department of Environmental Health Sciences, University of California, Berkeley, USA

## Abstract

**Background:**

Schistosomiasis infection, contracted through contact with contaminated water, is a global public health concern. In this paper we analyze data from a retrospective study reporting water contact and schistosomiasis infection status among 1011 individuals in rural China. We present semi-parametric methods for identifying risk factors through a comparison of three analysis approaches: a prediction-focused machine learning algorithm, a simple main-effects multivariable regression, and a semi-parametric variable importance (VI) estimate inspired by a causal population intervention parameter.

**Results:**

The multivariable regression found only tool washing to be associated with the outcome, with a relative risk of 1.03 and a 95% confidence interval (CI) of 1.01-1.05. Three types of water contact were found to be associated with the outcome in the semi-parametric VI analysis: July water contact (VI estimate 0.16, 95% CI 0.11-0.22), water contact from tool washing (VI estimate 0.88, 95% CI 0.80-0.97), and water contact from rice planting (VI estimate 0.71, 95% CI 0.53-0.96). The July VI result, in particular, indicated a strong association with infection status - its causal interpretation implies that eliminating water contact in July would reduce the prevalence of schistosomiasis in our study population by 84%, or from 0.3 to 0.05 (95% CI 78%-89%).

**Conclusions:**

The July VI estimate suggests possible within-season variability in schistosomiasis infection risk, an association not detected by the regression analysis. Though there are many limitations to this study that temper the potential for causal interpretations, if a high-risk time period could be detected in something close to real time, new prevention options would be opened. Most importantly, we emphasize that traditional regression approaches are usually based on arbitrary pre-specified models, making their parameters difficult to interpret in the context of real-world applications. Our results support the practical application of analysis approaches that, in contrast, do not require arbitrary model pre-specification, estimate parameters that have simple public health interpretations, and apply inference that considers model selection as a source of variation.

## Background

Schistosomiasis is a parasitic disease affecting an estimated 200 million people in 76 countries [1]. Humans become infected with schistosomiasis following contact with water containing cercaria, the larval stage of the parasite. Infection can lead to liver fibrosis and portal hypertension, and may cause anemia [2-4].

Recent studies have shown that the distribution of human schistosomiasis infections can be explained in part by spatial variability in water contact, particularly with respect to differences in cercarial density. For example, clusters of *Schistosoma hematobium *infections in rural Kenya were identified near water bodies with high numbers of cercaria-shedding snails [5]. Also, in contrast to water contact measures that ignore spatial variability in cercarial density, measures of water contact that adjust for estimated cercarial density at the site of contact have shown strong correlations with human infection intensity [6,7].

Less attention has been paid to temporal variability in infection risk and to the variability in infection risk from specific water contact activities. While diurnal variations in the infectivity of cercaria have been recognized for decades, little is known about the variability in infection risk throughout the transmission season [8]. Li *et al. *observed two annual peaks in *S. japonicum *infection prevalence in the lower Yantzee basin [9]. In the irrigated hillsides of southwest China, temporal fluctuations in both hydrology and snail populations have been documented, and may yield corresponding variation in infection risk throughout the transmission season [10,11]. Specific water contact activities may also affect infection risk, due perhaps to the location in which these activities are performed and the parts of the body exposed. Several specific water contact activities have been associated with the prevalence of *S. hematobium *infection in Zanzibar and *S. mansoni *infection in Cote d'Ivoire [12,13]. However, neither analysis accounted for the duration or timing of water contact, and such relationships have not yet been examined for *S. japonicum*.

The two studies of *S. mansoni *and *S. hematobium *mentioned above examined numerous risk factors for infection using traditional correlation and multivariate regression techniques. The multivariable regression approach, while common, imposes an arbitrary model that limits the interpretation of results [14]. For example, parameters from such models rarely have simply understood definitions within the context of the subject matter; they only have meaning within the context of the arbitrarily specified model. Multivariable regression models can also return misleading inference, because the assumption of an arbitrary model does not allow for model misspecification, and thus incorrectly estimates variability [15].

In contrast to multivariable regression, semi-parametric variable importance measures inspired by parameters from the causal inference literature have the virtue of (1) using machine learning algorithms to determine flexibly how to adjust for potential confounding variables without requiring arbitrary model pre-specification and (2) returning a simple and interpretable measure of variable importance that under assumptions can also yield estimates of the effect of intervention [16]. Such parameters have been referred to as population intervention parameters [16-19]. This alternative to a traditional regression analysis is well suited to the exploratory analysis of high-dimensional data, where one desires to investigate the independent association of one variable and an outcome in the presence of many correlated variables.

We analyzed data from a retrospective study in which 1011 individuals reported their water contact during the 2000 *S. japonicum *infection season in rural China; infection status in 2000 was also recorded for these individuals. Water contact was calculated using the estimated duration of water contact and the estimated body surface area in contact with water during the specific water contact activity. We aimed to explore the relative importance of different types of water contact, defined by both water contact activity and by the month in which the water contact occurred, on the probability of schistosomiasis infection. We analyzed these data in three ways: first, by applying a prediction (machine learning) algorithm; second, by using a simple multivariable regression; and third, by assessing variable importance using a causal inference-inspired population parameter. We discuss the results of each method, as well as the limitations of interpretation within the context of the method used.

## Methods

### Data Collection

This research was conducted in Xichang County located in the southwest of Sichuan Province, China. The region is hilly with irrigated agriculture and historically high schistosomiasis infection prevalence. Twenty villages ranging in size from approximately 100 to 300 residents were selected to participate in a cross-sectional study to characterize determinants of schistosomiasis infection [20]. In November 2000, all residents in the 20 villages were asked to participate in schistosomiasis infection surveys and in an interview to assess basic demographic characteristics including age, occupation and educational attainment. Participation rates were high: an estimated 90% of residents participated in these surveys. This research was conducted in close collaboration with the Xichang County Anti-Schistosomiasis Station and the Institute of Parasitic Diseases at the Sichuan Center for Disease Control. All participants provided verbal informed consent and human data collection protocols were approved by the Berkeley Committee for the Protection of Human Subjects and the Sichuan Institutional Review Board.

A 25% random sample of residents, stratified by village and occupation, was interviewed in person in November 2000 about their water contact patterns throughout the schistosomiasis transmission season. Participants were asked about eight different activities that involve contact with irrigation, pond or stream water each month from April through October: washing clothes or vegetables, washing agricultural tools, washing hands and feet, playing or swimming, irrigation ditch cleaning and water diverting, planting rice, harvesting rice and fishing. These water contact activities will be referred to subsequently as laundry, tool washing, bathing, swimming, ditch digging, rice planting, rice harvesting, and fishing, respectively. Participants were asked how often they performed each activity each month and for how many minutes each time, providing an estimate of water contact frequency and duration. Each activity was assigned an exposure intensity weight in order to account for differences in body surface area exposed. Field studies in the selected villages were conducted to observe which body parts were typically wetted for each water contact activity, and burn charts were used to estimate the percent of total body surface area accounted for in each exposed body part [21]. Water contact intensities were assigned as follows: laundry (0.05), tool washing (0.03), bathing (0.12), swimming (0.20), ditch digging (0.05), rice planting (0.05), rice harvesting (0.05) and fishing (0.32). Total body surface area for adults was estimated to be 1.626 m^2^, and for children age 14 and under: 1.130 m^2 ^[21]. For each activity *i *in month *k*, water exposure in minutes-meters^2 ^was calculated:

WCik=Frequencyik×Durationik×Intensityi×BodySurfaceArea.

An individual's water contact for each month was calculated by summing water exposure for all activities that month. Likewise, an individual's total water exposure for each activity was calculated by summing the activity-specific water exposure over the seven months. The total water contact over the entire period was also calculated. Because it was determined that only one infected individual had any water contact associated with rice harvesting, rice harvesting was excluded from the set of activity variables. This type of water contact was not excluded from the monthly water contact variables, or from the total water contact variables.

At the same time as the water contact surveys, and corresponding with the end of the transmission season, schistosomiasis infection surveys were conducted using two different stool examination techniques. Participants submitted stool samples from three different days and each sample was examined using the miracidial hatch test according to Chinese Ministry of Health protocols [22]. The Kato-Katz thick smear procedure was also used; three 41.5 mg slides were prepared from homogenized stool samples and examined for *S. japonicum *eggs [23]. Any person with a positive miracidial hatch test or at least one *S. japonicum *egg detected through Kato-Katz was classified as infected. All infected individuals were referred to local health officials for treatment with praziquantel.

### Statistical Analyses

#### *Prediction Algorithm*

In our first analysis, we used a machine-learning algorithm to choose the "best" set of infection predictors. This algorithm formed recursive partitioning, regression, and classification trees, as implemented in the R function *rpart *[24-26]. The algorithm was allowed to choose among all of the possible water contact variables, as defined above: activity type, water contact month, and total water contact. Since the activities are sums over all months, the months are sums over all activities, and the total is the sum of all water contact over the entire study period, including these variables together would not make sense in an approach attempting to determine associations between the variables and the outcome (as in the analyses conducted later in the paper). However, from the prediction standpoint, the only concern is the accuracy of prediction; it makes the most sense, therefore, to include as many variables as possible in the potential prediction algorithm, which is why we included all variables. We note that *rpart *is just one of many machine learning algorithms that could be used, including algorithms that combine results from several learners [27]. This approach generalizes to any such routines.

In an attempt to assess the relative "importance" of the variables in predicting the outcome, we applied a Monte Carlo re-sampling approach (nonparametric bootstrap) [28]. The study individuals were randomly re-sampled with replacement (meaning that one subject could be sampled more than once, but that all samples were of the same size), and the *rpart *tree was recalculated. This bootstrapping method is a commonly used way of simulating re-sampling from the target population, and can help to examine how small changes in the data can affect the prediction model chosen. We performed this re-sampling approach 5000 times, and tabulated the number of times each variable was chosen by *rpart *in the prediction model. Multiple splits on a given variable within the same *rpart *fit were counted only once on each iteration.

#### *Multiple Regression*

Turning away from the prediction-focused approach, our second analysis was a main-effects log-linear regression, in which we also included age category (< 18, 18-29, 30-29, 40-49, 50+) and village indicator variables as possible confounders. Here we separated the activity types from the months into two separate models, and excluded total water contact from both models. We could not use log-linear binomial models because they generated predicted probabilities that exceeded one, so we used instead Poisson log-linear models.

Model 1:log[E(Y|Wactivity,V)]=α+βactivityWactivity+γV

Model​​ 2:log[E(Y|Wmonth,V)]=α+βmonthWmonth+γV

In both models, *Y *is the (binary) outcome, *V *is the vector of village and age category indicators, and *γ *is the vector of coefficients associated with *V*. In Model 1, *W*_*activity *_is the vector of activity type water contact variables, and *β*_*activity *_is the vector of activity type coefficients; in Model 2, *W*_*month *_is the vector of monthly water contact variables, and *β*_*month *_is the vector of month coefficients. Because we did not wish to rely upon the Poisson assumption for estimating our standard errors and deriving inference, we instead calculated robust standard errors using the Huber/White sandwich estimator [29,30]. Regression estimates were obtained using the *glm *command in Stata [31].

#### *Variable Importance*

Our third (semi-parametric) approach estimated a so-called *variable importance *(VI) parameter which compares the current distribution of the outcome to its distribution under a theoretical experiment where the variable of interest is set to the lowest risk. In our data, this is equivalent to comparing the observed infection prevalence distribution to the distribution of infection in a theoretical experiment in which the entire study population never experienced a particular type of water contact.

Assume the current variable of interest is *A*, the outcome is *Y*, and the confounders - in this case, all other water contact variables except *A *- are *W*, and *V *are the additional confounders (age category and village). Our VI estimate is inspired by the following causal parameter:

$$\frac{E(Y_{0})}{E(Y)}.$$

*Y*_*a *_represents the outcome if - possibly contrary to fact - everyone had exposure *A = a*. (Outcomes defined in such a way have been referred to as *counterfactuals *[32].) In the case of our binary outcome variable, *E(Y) *is estimated as the current disease prevalence in our target population, which is estimated as the average of the observed *Y *values.

If *Y *is binary (yes/no) - as it is in our case - this parameter can be interpreted as the proportional change, relative to current rates, in the prevalence of schistosomiasis in our target population if everyone were unexposed to the particular risk. This parameter is akin to the attributable risk, and its magnitude is both a function of the adjusted association of *A *and *Y *and of the prevalence of exposure. For example, removing exposure would have little effect on the value of this causal parameter if the exposure in question were very rare, even if it were strongly related to the disease outcome. Conversely, removing a common exposure that only modestly increased the risk of disease could have a much larger impact on the parameter's value.

With regards to the distribution of the data alone - that is, without assuming the necessary identifiability conditions for making causal inference (no unmeasured confounders and independence of counterfactual outcomes, or the so-called stable unit treatment value assumption - SUTVA [33]) - our VI measure is an estimate of the following:

$$VI=\frac{E_{W,V}E(Y|A=0,W,V)}{E(Y)}.$$

The numerator is interpreted as the mean predicted value of *Y *assuming one sets the exposure to 0 (*A *= 0 means unexposed) but keeps the other variables at their observed values. *E*_*W, V *_in the numerator denotes that this mean predicted value of *Y *is also taken over all *W *and *V*.

The denominator was estimated by simply taking the mean of the *Y *values. To estimate the numerator, we used the so-called inverse-probability-of-censoring-weighted (IPCW) estimator:

$${\hat{E}}_{W,V}\hat{E}(Y|A=0,W,V)=\frac{1}{n}\sum_{i=1}^{n}\frac{I(A_{i}=0)Y_{i}}{\hat{P}(A_{i}=0|W_{i},V_{i})}.$$

Here $\hat{P}(A_{i}=0|W_{i},V_{i})$ is an estimate of the probability that *A *= 0 given the values of the covariates *W_i _*and *V*_*i *_for subject *i*. The form of this estimator makes obvious another assumption, which has been called positivity or experimental treatment assignment (ETA) assumption, which in this case says that *P(A = 0|W, V) > 0 *in the data-generating distribution [34-36].

The IPCW estimator is a type of weighted average of the *Y *values, in which the weights are proportional to the probability of being unexposed (*A_i _= 0*) given the other covariates (*W*_*i *_and *V*_*i*_). The IPCW estimator relatively up-weights the disease outcomes of unexposed individuals with covariates underrepresented within the unexposed group, which has the effect of adjusting for confounding bias. Because *P(A_i_|W_i_, V_i_) *is unknown in this case, we used a machine-learning algorithm (*rpart*) to estimate a model for this probability.

A VI estimate was calculated for each variable of interest. Specifically, we define the VI estimate for each water contact activity as follows:

$$VI_{activity}=\frac{E_{W_{activity},V}E(Y|A=0,W_{activity},V)}{E(Y)},$$

where *A *represents the water contact activity type for which a VI estimate is being calculated, *W*_*activity *_represents the remaining water contact activity type variables, and *V *represents the age category and village covariates. The VI estimate for each month is defined equivalently, with *W_month _*in place of *W*_*activity*_. As in the logistic regression analysis, total water contact was excluded; it would not be meaningful to estimate *E_W, V _E*(*Y*|*A *= 0, *W*, *V*) for *A = *total water contact, since none of the other water contact variables could be nonzero if total water contact were equal to zero.

To derive our inference, we estimated standard errors using the non-parametric bootstrap with 5000 iterations. Specifically, participants were re-sampled with replacement, producing 5000 bootstrap samples of size 1011. For each of these 5000 samples, VI estimates were calculated, including a re-calculation of $\hat{P}(A_{i}=0|W_{i},V_{i})$. The standard deviation across these 5000 estimates was then calculated and used for inference. Because the model for *P*(*A*_*i *_= 0|*W*_*i*_, *V*_*i*_) was not pre-specified, this method of calculating the standard error will account for both sampling variability (by re-sampling) and the variability introduced by model uncertainty with regards to *P*(*A_i _*= 0|*W*_*i*_, *V*_*i*_) (by allowing for changes in the model for $\hat{P}(A_{i}=0|W_{i},V_{i})$ at each iteration).

## Results

**Figure 1:**
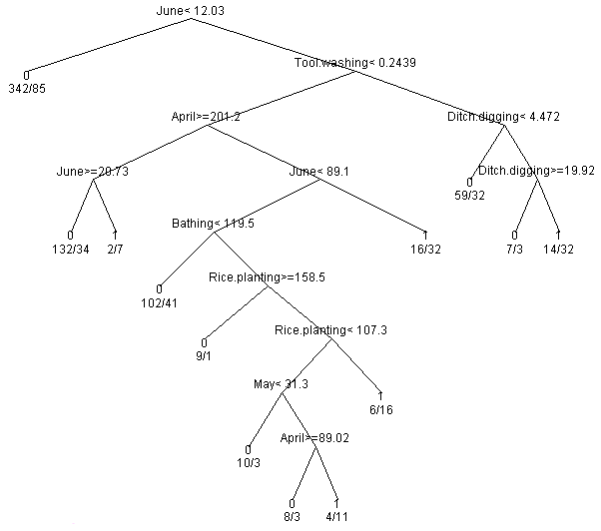
**Full data *rpart *classification tree**.

Figure 1 shows the full data *rpart *tree formed by allowing the machine learning algorithm to choose splits from the pool of all water contact variables. April, May, June, tool washing, ditch digging, bathing, and rice picking were the water contact variables chosen for classification.

When the data were re-sampled with replacement, Table 1 lists the number and percentage of times (out of 5000) each variable was chosen for classification in a given *rpart *tree. The covariates are ordered according to the number of times they were chosen to be part of each *rpart *tree, from largest to smallest. This method identified April (92%), June (92%) and total water contact (86%) as the most frequently chosen predictors of infection status within the bootstrapping algorithm. The six variables chosen for classification in the original full data tree (Figure 1) are among the top seven identified most frequently for use in the bootstrap sample *rpart *trees. However, total water contact, chosen 86% of the time in the bootstrap samples, was not part of the original full data tree.

**Table 1:** Number of times out of 5000 that each water contact type was chosen by *rpart *to form a data-adaptive classification tree.

Water contact type	Number of times chosen	Percentage
April	4608	92.2%
June	4602	92.0%
Total	4283	85.7%
Tool washing	4067	81.3%
Ditch digging	3825	76.5%
Rice planting	3677	73.5%
May	3652	73.0%
September	3326	66.5%
July	3181	63.6%
Bathing	3073	61.5%
October	2787	55.7%
Swimming	2481	49.6%
Laundry	2133	42.7%
August	1892	37.8%
Fishing	69	1.4%

Data to form each tree were obtained by re-sampling the 1011 individuals with replacement.

Tables 2 and 3 show results from the log-linear regression models, along with the prevalence of each type of water contact in our sample. The correlations between the various water contact variables range from -0.02 (between April and August) to 0.68 (between July and August) for the monthly variables and from -0.15 (between swimming and bathing) and 0.28 (between rice picking and bathing) for the activity variables. The reported relative risks were calculated as $e^{{\hat{\beta }}_{i}{\bar{X}}_{i}}$, where ${\hat{\beta }}_{i}$ is the estimated regression coefficient and ${\bar{X}}_{i}$ is the mean water contact across all subjects for water contact variable *i*. This relative risk therefore reports the risk of having the mean value for water contact variable *i *versus the risk of having no water contact of type *i*. As previously mentioned, the month and activity variables were separated into two different models, which is why the results are reported separately. The estimates in Tables 2 and 3 are also adjusted for age category and village. We do not report relative risks associated with age category and village because the effects of these covariates were not the focus of this study.

**Table 2:** Relative risk estimates for water contact by month.

Month	Prevalence	Relative Risk	95% CI	Std. error	p-value
June	0.75	1.03	(0.98, 1.09)	0.03	0.20
October	0.58	0.95	(0.89, 1.03)	0.04	0.22
May	0.75	1.04	(0.97, 1.13)	0.04	0.25
April	0.73	1.04	(0.95, 1.14)	0.05	0.45
August	0.76	1.03	(0.94, 1.13)	0.05	0.51
September	0.70	0.98	(0.89, 1.07)	0.05	0.64
July	0.77	0.98	(0.91, 1.06)	0.04	0.68

These estimates are based on a main-effects log-linear regression, and are also adjusted for age category and village. The relative risks reflect the difference in risk of infection between exposure at the mean value for that month and zero exposure.

**Table 3:** Relative risk estimates for water contact by activity.

Month	Prevalence	Relative Risk	95% CI	Std. error	p-value
Tool washing	0.20	1.03	(1.01, 1.05)	0.01	< 0.01
Laundry	0.22	1.02	(1.00, 1.05)	0.01	0.08
Swimming	0.21	1.02	(1.00, 1.05)	0.01	0.10
Ditch digging	0.48	0.99	(0.98, 1.00)	0.01	0.16
Fishing	0.02	1.01	(1.00, 1.02)	0.01	0.17
Bathing	0.49	0.98	(0.92, 1.04)	0.03	0.46
Rice planting	0.65	1.03	(0.94, 1.13)	0.05	0.52

These estimates are based on a main-effects log-linear regression, and are also adjusted for age category and village. The relative risks reflect the difference in risk of infection between exposure at the mean value for that month and zero exposure.

In the log-linear regression framework, none of the monthly water contact variables were found to have strong associations with the outcome. All month-specific relative risk estimates are very close to one and have 95% confidence intervals that include one. This implies that the risk of having a positive stool sample when these variables are at their mean values is indistinguishable from the risk when there is zero water exposure during these months. Similarly, the relative risks associated with the water contact activity types are also all very close to one, and almost all have 95% confidence intervals that include one. The tool washing-specific relative risk has a 95% confidence interval that does not cross one; the estimated relative risk is still extremely close to one, however, implying almost no detected difference in risk. These results are of course only interpretable in the context of the regression models used.

Tables 4 and 5 show VI estimates for the two sets of water contact variables. As in the log-linear regression framework, the monthly water contact variables were analyzed separately from the water contact activity variables. As previously explained, the VI estimates were adjusted for age category and village by including these variables in the estimation of *P(A_i_|W_i_, V_i_)*. (In similarity with the regression analysis, we did not calculate VI estimates for age category and village.) Confidence intervals and *p*-values based on the bootstrap-derived standard errors are also reported. In contrast to the log-linear regression results, which identified no detectable adjusted associations with the outcome among the monthly water contact variables, July's VI estimate indicates a strong adjusted association. If one interprets this VI estimate as an estimate of $\frac{E(Y_{0})}{E(Y)}$, it implies that eliminating water contact in July would reduce the prevalence of schistosomiasis measured in the study by 84%, or from 0.3 to 0.05. The 95% confidence interval for this estimate indicates a range of 78% to 89%. The prevalence of exposure in July is 0.77, which along with August is the highest of any month. The VI estimates for all other months are near one and have 95% confidence intervals that include one (many of which are quite broad). No other month, therefore, has a detectable association with the outcome.

**Table 4:** Variable importance estimates for water contact by month.

Month	Prevalence	VI estimate	95% CI	Std. Error	p-value
July	0.77	0.16	(0.11, 0.22)	0.18	< 0.01
August	0.76	1.70	(0.48, 6.02)	0.66	0.42
May	0.75	1.18	(0.32, 4.30)	0.66	0.81
October	0.58	1.05	(0.60, 1.84)	0.28	0.86
June	0.75	0.97	(0.27, 3.56)	0.66	0.97
September	0.70	1.01	(0.41, 2.49)	0.46	0.98
April	0.73	1.00	(0.40, 2.50)	0.46	1.00

The prevalence of water contact for each month in our study population is also shown.

**Table 5:** Variable importance estimates for water contact by activity type.

Month	Prevalence	VI estimate	95% CI	Std. Error	p-value
Tool washing	0.20	0.88	(0.80, 0.97)	0.05	0.01
Rice planting	0.65	0.71	(0.53, 0.96)	0.15	0.03
Swimming	0.21	0.96	(0.87, 1.06)	0.05	0.38
Ditch digging	0.48	0.94	(0.80, 1.10)	0.08	0.42
Bathing	0.49	1.09	(0.88, 1.35)	0.11	0.42
Laundry	0.22	0.97	(0.89, 1.06)	0.04	0.45
Fishing	0.02	1.00	(0.98, 1.02)	0.01	0.83

The prevalence of water contact for each month in our study population is also shown.

In terms of VI, no other type of water contact had as large an impact on infection risk as July water contact. Tool washing and rice planting were the only two activities with a discernable impact on infection risk - all other activity types (Table 4) have VI estimates near one and 95% confidence intervals that include one. Both of the VI estimates associated with tool washing and rice planting, in contrast, have 95% confidence intervals that do not cross one. Interpreting the VI results once again as estimates of $\frac{E(Y_{0})}{E(Y)}$ would imply an estimated 12% reduction in the prevalence of schistosomiasis by eliminating tool washing and an estimated 29% reduction by eliminating rice planting. The associated 95% confidence intervals for these estimates imply a range of 3% to 20% for tool washing and 4% to 47% for rice planting. As shown in Table 5, the prevalence of water exposure due to tool washing in our study population was 0.20, while the prevalence of water exposure due to rice planting was 0.65.

## Conclusions

The three analysis approaches used here are all attempts to answer the same research question: what is the best estimate of the contribution of one explanatory variable to the mean outcome in the presence of other correlated explanatory variables? We specifically hoped to see how various types of water contact affected the probability of a positive stool sample, adjusting for other types of water contact, age, and village.

The use of machine learning algorithms for model selection is attractive, particularly because the model does not have to be pre-specified; this means estimating the association parameters while acknowledging that very little is typically known about the form of the model. A comparison of Figure 1 and Table 1, however, provides an example of how simply determining whether or not a variable is chosen by a machine learning algorithm (such as *rpart*) is not a particularly robust procedure for defining the importance of a variable. Given a finite sample size and highly correlated predictors - as we have in our data - small changes in the data often result in large changes in the variables chosen as predictors. This can occur even as the fidelity of prediction is nearly unchanged; there are often several sets of variables in various functional forms that can provide nearly identical accuracy of prediction. This issue is partially what inspired the idea of bagging or bootstrapping these machine learning algorithms, such as in the case of random forests [37]. For example, our full data tree could lead us to conclude that total water contact is less predictive of a positive stool sample than the specific activity and month variables chosen to be part of the tree. Table 1, however, would lead us to conclude that total water contact is one of the top three most predictive variables - and therefore more "important" than four out of the six variables identified in the full data tree. Due to this instability, machine learning algorithms alone provide sub-optimal information for determining the importance of variables.

The actual best set of predictor variables is a function of the type of model, the method for constructing candidate models, and the method used to choose the so-called tuning parameters. Our results here therefore do not generalize to all machine learning routines - such as, for example, the Deletion/Substitution/Addition algorithm [38], POLYCLASS [39] or random forests [37]. Generally, as implied by the results displayed in Table 1 and Figure 1, prediction algorithms are not constructed to provide any easily interpretable estimates of each water contact variable's contribution to the probability of a positive stool sample, which is ultimately what we were trying to investigate. Machine learning algorithms can be applied most effectively to answering our question of interest when used within an estimation framework whose parameters are defined independently from the specific model chosen by a given algorithm (such as *rpart*). This semi-parametric approach, of which our VI analysis is an example, contrasts dramatically with estimating simple, parametric regression models and reporting the resulting coefficients as association parameters (such as the relative risks reported in Tables 2 and 3). Though such regression analyses can produce parameters with relatively straightforward public health interpretations, the interpretations only remain straightforward if the pre-specified regression model is correct; any interpretation of the estimates obtained must implicitly assert the truth of the model used, though there is very rarely any justification for a specific parametric model's *a priori *truth. In addition, the lack of data-adaptive procedures can sacrifice power by resulting in much larger residual variability than approaches that use the data to fit the models. Tables 2 and 3, for example, show that under the constraints of the regression model, even the coefficients with 95% confidence intervals that did not cross one yielded relative risks very close to one, suggesting little contribution to the variability of the outcome. Whether this is a true result, however, or merely reflective of a poorly chosen model, is impossible to assess. The regression approach, though common, is therefore a dangerous choice as a basis for making causal inferences. Interpretation of parameters (conditional relative risks) in the context of a misspecified model are also of dubious value, since it is difficult to know what such interpretations really mean. This is true of the innumerable regression approaches reflexively used throughout observational epidemiology and other empirical fields.

Though one data analysis cannot justify the global use of an analysis technique, at least there is some hope that our approach here has found potentially interesting associations. Specifically, the importance of July water contact in our VI results - not detected by the regression analysis - could suggest temporal variability in infection risk during the infection season. This could be due to a combination of factors, since infection risk depends not only on water contact intensity but also on cercarial concentration in that water. A summer peak in cercarial concentration was observed in a number of villages in this same area in 2001 using a mouse bioassay procedure throughout the infection season [40]. The peak occurred in August, not July, but year-to-year variability in cercarial concentration can be expected due to seasonal fluctuations in snail populations and agricultural activities driven by changes in rainfall, temperature, and humidity. Temporal variability in infection risk can also be influenced by seasonal changes in activities known to be associated with infection, such as swimming, which may increase during summer months when school is not in session and ambient temperatures are high. In addition, prior work has documented seasonal fluctuations in hydrology which correspond to differences in infection patterns between schistosomiasis endemic regions within Sichuan province [11]. One must consider, however, that this dataset has a number of limitations. The retrospective nature of the water contact surveys calls into question the accuracy of recall - particularly given the relatively long period of time (seven months) during which study participants were asked to recount their water contact activities. The analysis also relies on the definition of water contact, which as previously described includes an estimate of the body surface area believed to be in contact with water during certain activities. We are additionally limited by the need to analyze the monthly water contact and water contact activity variables separately; while it would have been ideal to consider the 56 activity type-by-month variables, the number of covariates is simply too large in comparison with the sample size for any technique to single out individual contributions. We therefore chose to simplify the set of variables by considering activity separately from month, thus providing some power to detect adjusted associations.

While the results of this analysis are far from conclusive, they nonetheless suggest possibly fruitful areas for future research. If a high-risk period in the schistosomiasis infection season could be detected in something close to real time, new prevention options would be opened. Recent advances in detecting schistosome cercariae in water using PCR techniques could potentially provide such a tool [41]. The notion of changing from a surveillance system that relies on episodic human infection surveys to one based on water monitoring has many attractions, including the likelihood of lower cost. Water monitoring is also an appealing option in areas where schistosomiasis re-emergence has occurred or is suspected [42].

Though we compare here three specific analysis techniques, we note that many different machine learning algorithms (other than classification trees) are available, different regression models could be specified, and different approaches to estimating our VI parameter could be used (including G-computation and Targeted Maximum Likelihood) [43,44]. The general principals contrasting these methods remain the same, however, and are important in the larger issue of estimating the independent and potentially causal association of risk factors in data sets with large numbers of covariates. Prediction (machine learning) algorithms are very well-designed to provide optimal prediction and to balance the variance and bias in the predicted value (the estimate of *E(Y|A, W, V)*); they are not optimal for determining the contributions of individual variables directly. This is particularly obvious since small changes in the data can result in large changes in the variables chosen. In contrast, the standard regression model approach has a nicely interpretable parameter, but is entirely dependent upon the correctness of the model specified. The definition of the parameter itself is also generally tied to the form of the model - for example, adding a multiplicative interaction term into a regression model changes the meaning of the main effect term. Thus, the definition of a given parameter is only useful if the model is correct, and that parameter's interpretation changes as other variables are added to or removed from the model. In reality, such models are never correct, and there is no mechanism for allowing them more flexibility (such as through machine learning algorithms) to reduce bias as sample size grows. These issues expose the need for a meaningful parameter, one whose estimation can capitalize on the virtues of the asymptotic bias-reduction of machine learning algorithms and whose definition is not dependent upon the model chosen by these algorithms. The VI parameter we use is an answer to this need. We employ a machine learning algorithm to estimate the parameter, but differences in the model chosen by the algorithm do not change the definition of the parameter.

The semi-parametric approach is evolving, and recent advances promise to increase the power of this combination of machine learning and causal inference methods. We do not necessarily advocate the details of the semi-parametric VI algorithm used here - we in fact used a relatively inefficient method, and more refined methods are available to target model selection towards optimizing the particular parameter of interest [45]. We simply argue that it is possible to devise estimation strategies that, given unavoidable assumptions, can converge to unbiased estimates of the causal effects defined as sample size grows. In addition to the aforementioned alternate approaches for estimating our VI parameter, one can also use so-called asymptotically linear estimators; these are normally distributed, and in many cases simple standard errors based on this normality can be derived if one wishes to avoid re-sampling-based techniques (i.e. the bootstrap).

Risk factor epidemiology has for too long relied upon inherently biased techniques, particularly for observational data. There is no longer any reason to do so; the bias-reduction flexibility of semi-parametric models can be combined with estimation of simple and frankly more meaningful parameters in public health. We suggest using techniques that (1) define parameters with convenient public health interpretations, (2) use flexible, data-adaptive routines that do not pre-suppose arbitrary and scientifically unjustifiable models, and (3) employ honest inference that accounts for all the aspects of variation, including model selection.

## Competing interests

The authors declare that they have no competing interests.

## Authors' contributions

SS primarily conducted the analyses, helped with writing and edited the paper. EC helped with writing the Background, and EC and ES helped interpret the results in the context of their study in China. RS helped to write the Conclusions and put the methodological contribution of the paper in the context of the methods used for these type of infectious disease studies. AH helped to derive the methods, conduct the regression analyses, and write/edit the paper. All authors read and approved the final manuscript.
